# Skin Lesions Associated with Nutritional Management of Maple Syrup Urine Disease

**DOI:** 10.1155/2017/3905658

**Published:** 2017-10-25

**Authors:** Jaraspong Uaariyapanichkul, Puthita Saengpanit, Ponghatai Damrongphol, Kanya Suphapeetiporn, Sirinuch Chomtho

**Affiliations:** ^1^Division of Nutrition, Department of Pediatrics, Faculty of Medicine, Chulalongkorn University, Bangkok 10330, Thailand; ^2^Division of Nutrition, Department of Pediatrics, King Chulalongkorn Memorial Hospital, The Thai Red Cross Society, Bangkok 10330, Thailand; ^3^Center of Excellence for Medical Genetics, Department of Pediatrics, Faculty of Medicine, Chulalongkorn University, Bangkok 10330, Thailand; ^4^Excellence Center for Medical Genetics, King Chulalongkorn Memorial Hospital, The Thai Red Cross Society, Bangkok 10330, Thailand

## Abstract

**Introduction:**

Maple syrup urine disease (MSUD) is an inborn error of branched chain amino acids (BCAAs) metabolism. We report an infant with MSUD who developed 2 episodes of cutaneous lesions as a result of isoleucine deficiency and zinc deficiency, respectively.

**Case Presentation:**

A 12-day-old male infant was presented with poor milk intake and lethargy. The diagnosis of MSUD was made based on clinical and biochemical data.

**Management and Outcome:**

Specific dietary restriction of BCAAs was given. Subsequently, natural protein was stopped as the patient developed hospital-acquired infections which resulted in an elevation of BCAAs. Acrodermatitis dysmetabolica developed and was confirmed to be from isoleucine deficiency. At the age of 6 months, the patient developed severe lethargy and was on natural protein exclusion for an extended period. Despite enteral supplementation of zinc sulfate, cutaneous manifestations due to zinc deficiency occurred.

**Discussion:**

Skin lesions in MSUD patients could arise from multiple causes. Nutritional deficiency including isoleucine and zinc deficiencies can occur and could complicate the treatment course as a result of malabsorption, even while on enteral supplementation. Parenteral nutrition should be considered and initiated accordingly. Clinical status, as well as BCAA levels, should be closely monitored in MSUD patients.

## 1. Introduction

Maple syrup urine disease (MSUD; OMIM# 248600) is an autosomal recessive inborn error of metabolism, which can be managed by specific dietary modifications of branched chain amino acid intake. The incidence of MSUD is approximately 1 in 185,000 worldwide [1]. Although each disorder of inborn errors of metabolism is individually rare, their cumulative incidence is substantial (an incidence of 1 in 2,500–5,000 live births) and has been shown to be upwards of 1 in 800 [2].

We report an infant with MSUD who developed cutaneous lesions as a result of isoleucine deficiency while on dietary management. The skin lesions resolved after addition of isoleucine to the diet with normalization of isoleucine levels. We also report subsequent cutaneous lesions as a result of zinc deficiency during the following episode of treatment.

## 2. Case Report

A 12-day-old male Indian infant was referred to a tertiary care hospital due to poor milk intake and lethargy. The patient was a full-term neonate with an uncomplicated delivery, without any family history of metabolic disorder or consanguinity. The disease started 3 days earlier and the infant was previously admitted to a private hospital and received intravenous antibiotics for the treatment of presumed neonatal sepsis, but without any improvement. Initial biochemical investigations showed ketonuria, no metabolic acidosis, normoglycemia, and a mildly elevated level of ammonia (125 *μ*mol/L). Microbiological examinations were all negative. Upon arrival, the patient had seizure and was intubated.

Further biochemical study with plasma amino acid analysis showed a leucine level of 4163.6 *μ*mol/L (reference values: 42–133.1 *μ*mol/L), isoleucine of 499.8 *μ*mol/L (15.1–74.9 *μ*mol/L), and valine of 784.3 *μ*mol/L (73.6–273.1 *μ*mol/L). Thus, the diagnosis of MSUD (maple syrup urine disease) was made based on clinical and biochemical data.

**Figure 1:**
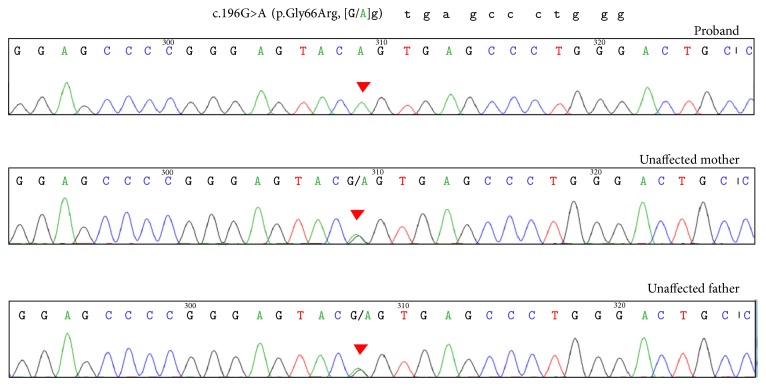
Electropherogram of the proband and his parents.

Whole exome sequencing revealed a novel homozygous missense variant c.196G>A (p.Gly66Arg) in the branched chain keto acid dehydrogenase E1 subunit beta* (BCKDHB)* gene. The variant was confirmed by PCR-Sanger sequencing to be homozygous in the patient and heterozygous in both parents (Figure 1).

Initial treatment included intravenous fluid and glucose with the aim to provide adequate energy to reduce catabolism and cessation of protein intake for 48–72 hours, administration of cofactors including thiamine, and adjunct treatment of neurological complications. Specific dietary restriction of branched chain amino acids (BCAAs) by using a branched chain-free amino acid supplement was given at the age of 17 days together with expressed breast milk as a source of natural protein via nasogastric tube.

**Figure 2:**
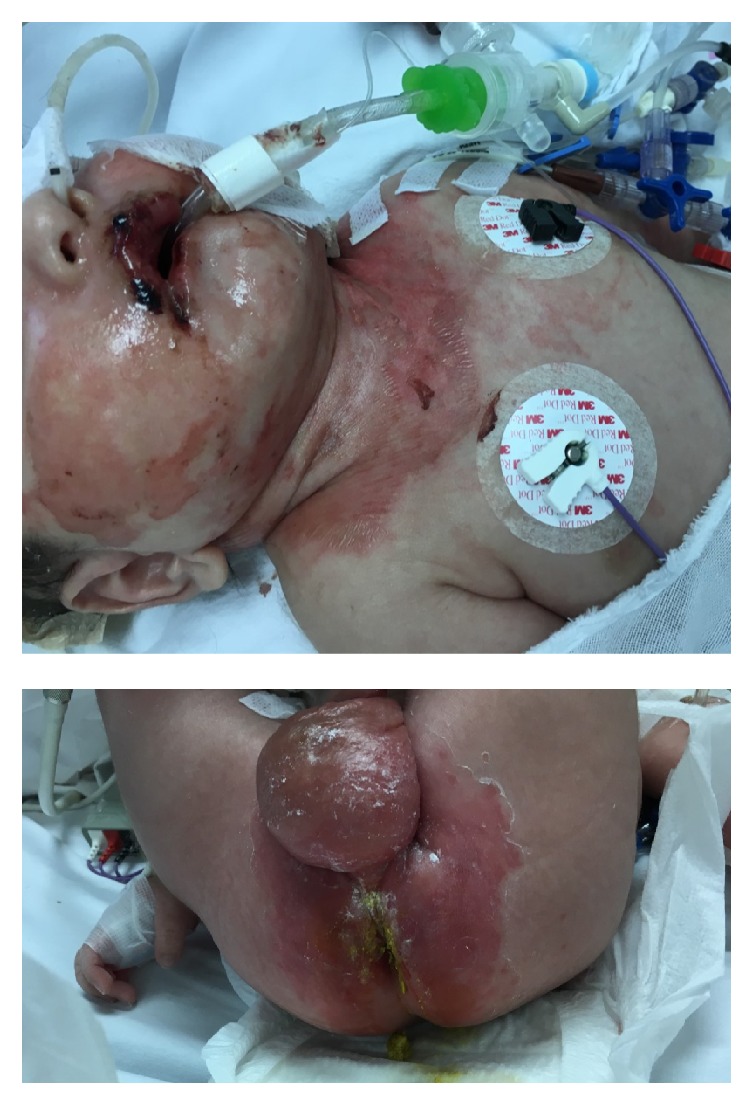
Skin lesions in the perioral, cheeks, neck, upper anterior chest wall, and perianal regions seen in an MSUD infant with isoleucine deficiency.

On day 36, natural protein was stopped as the patient developed hospital-acquired pneumonia and diarrhea which resulted in an elevation of BCAAs. On day 45, multiple discrete erythematous macules and papules appeared on the skin of the forehead, hemorrhagic crusts at both upper and lower lips, as well as well-defined erythematous patches at both cheeks, flexor part of neck, forearms, upper anterior chest wall, and perianal regions which rapidly progressed (Figure 2).

The amino acid profile revealed an isoleucine level of 5.9 *μ*mol/L, leucine of 1166 *μ*mol/L, and valine of 159 *μ*mol/L. The serum zinc level was normal (74 *μ*g/dL). Mycological and microbiological examinations were performed and showed negative results. A final diagnosis of acrodermatitis dysmetabolica due to isoleucine deficiency was made based on the clinical findings and low isoleucine levels through this period. Expressed breast milk was added to the feeds with isoleucine supplementation of 100 mg/day. The patient had been on continuous venovenous hemofiltration for a 48-hour period to reduce elevated leucine levels. In addition, skin care with emollients was administered. The patient showed a rapid improvement in the skin lesions which started to recover within 2 days and healed in 10 days with no hyperpigmentation. Subsequent amino acid profile showed an increment of isoleucine to a normal level (45.97 *μ*mol/L).

After hospital discharge, the feeding regimens were continued with branched chain-free amino acid supplement and expressed breast milk with supplementation of isoleucine 100 mg/day and valine 50 mg/day to prevent the deficiency, which yielded total energy of 130 kcal/kg/day, total protein of 3 g/kg/day, leucine, isoleucine, and valine of 50 mg/kg/day each.

**Figure 3:**
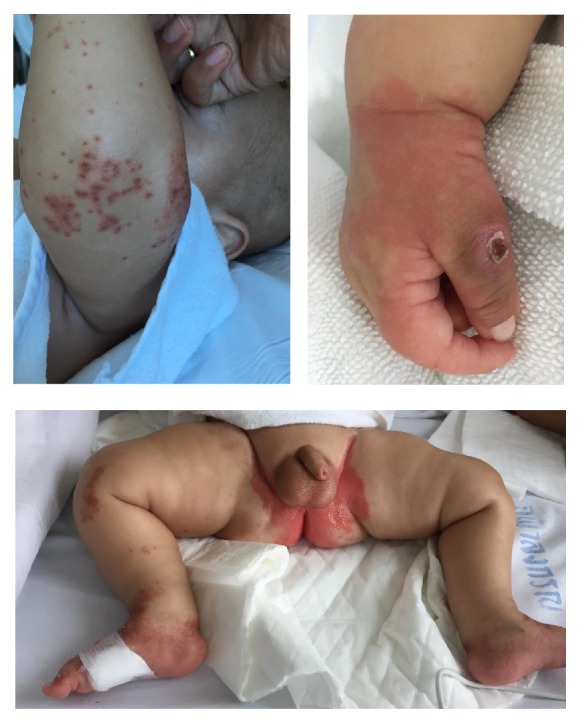
Skin lesions on the extremities and perianal region seen in an MSUD patient with zinc deficiency.

A subsequent episode of cutaneous manifestations occurred at the age of 6 months. The patient developed severe lethargy. Leucine encephalopathy was initially suspected and dietary management with cessation of natural protein intake was started as a part of the MSUD emergency protocol. Despite receiving seemingly adequate energy (110 kcal/kg/day) and protein intake from branched chain-free amino acid supplement (3 g/kg/day), the patient's level of consciousness was slow to improve and he was on natural protein exclusion for an extended period despite continuing isoleucine and valine supplementation. A complication of parainfluenza infection and a significant period of diarrhea soon followed and he had been on enteral supplementation of zinc sulfate 2 mg/kg/day together with other micronutrients supplementation. Ten days after admission, disseminated brown maculopapular exanthem appeared on the skin of the extremities, as well as well-defined erythematous patches on the perianal region (Figure 3).

Isoleucine deficiency was initially suspected; however, the amino acid profile later still showed elevated levels of branched chain amino acids with leucine of 533.25 *μ*mol/L (41.63–189.6 *μ*mol/L), isoleucine of 490.53 *μ*mol/L (18.37–72.26 *μ*mol/L), and valine of 400.05 *μ*mol/L (78.57–263.66 *μ*mol/L). Hence, in this subsequent episode of cutaneous manifestation, zinc deficiency causing acrodermatitis enteropathica was confirmed as the serum zinc level was low (56.9 *μ*g/dL) despite continued supplementation.

**Figure 4:**
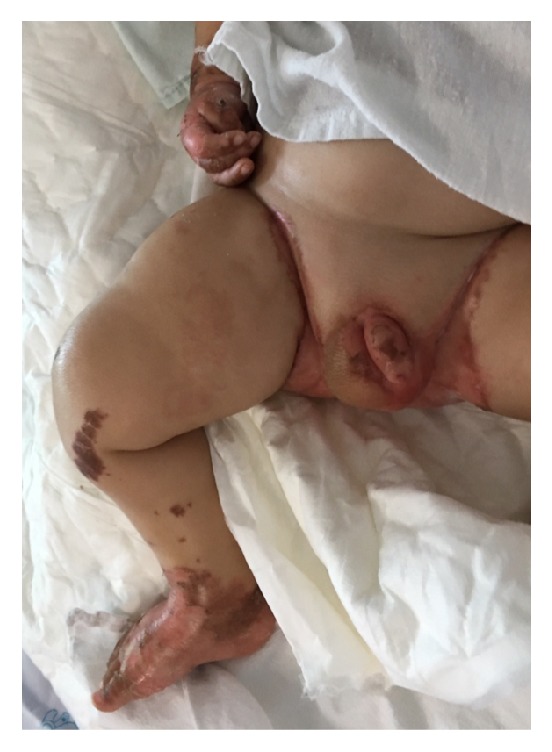
The patient became edematous with skin desquamation in the extremities and perianal region during the course of treatment.

Skin lesions showed no significant improvement after an increased dosage of enteral chelated zinc to 4 mg/kg/day. Parenteral nutrition with added zinc was then given intravenously due to suspected malabsorption. However, the skin lesions continued to worsen with desquamation in the following week. The patient became edematous, also suspected to be from protein deficiency as a result of malabsorption during the course of treatment (Figure 4). Albumin transfusion, parenteral nutrition containing limited amount of amino acids (based on leucine intake), and extra zinc were given along with branched chain-free amino acid supplement and led to his recovery.

## 3. Discussion

Dietary restriction of BCAAs (leucine, valine, and isoleucine) while avoiding their deficiency, maintaining growth and nutritional status, and avoiding catabolism is the mainstay of nutritional therapy in MSUD [3]. The aim is to maintain plasma BCAA concentrations within a target treatment range that is not associated with neurotoxicity [3].

The patient developed isoleucine deficiency and presented with skin lesions of acrodermatitis dysmetabolica. These cutaneous lesions have been described during treatment of aminoacidopathies (MSUD) [4, 5] and organic acidemias (methylmalonic acidemia, glutaric aciduria, and propionic acidemia) [6, 7] due to deficiency of isoleucine. It has been called acrodermatitis acidemica [6–10] or acrodermatitis dysmetabolica [10, 11]. Other nutritional causes have also been described such as biotin and free fatty acid deficiency [12]. Moreover, inadequate intake of BCAAs can also induce exfoliative erythroderma in MSUD infants [13].

In Thailand, MSUD patients have been diagnosed; however, there is no report in details regarding the complication of skin manifestations. Our patient had the clinical profile and responded to treatment similar to the cases with isoleucine deficiency described previously. Treatment options to prevent deficiency of branched chain amino acids include supplementation with specific amino acid powder, supplementation of expressed breast milk, or infant formula with known amino acid concentration as sources of natural protein. It is also necessary to frequently monitor the patients to optimize their growth and dietary intake and ensure normal levels of branched chain amino acids.

During the treatment course, skin lesions can develop. It is essential to be aware of their causes such as zinc deficiency and specific amino acid deficiency despite enteral supplementation. Malabsorption can occur, especially during and after an episode of diarrhea or infection. For this patient, parenteral nutrition is delayed during the turnaround time for laboratory reports of plasma amino acids and zinc which were not readily available in Thailand. We believe that this scenario is still pertinent with other developing countries as well. Nevertheless, we do hope that, by learning from this case, caregivers will become more aware of these complications and may lead to better care for patients with inborn errors of metabolism, especially regarding earlier initiation of parenteral nutrition.

## 4. Learning Points

- Skin lesions in MSUD patients could arise from multiple causes.
- Nutritional deficiency including isoleucine, zinc, and protein deficiency can occur and could complicate the treatment course, even while on enteral supplementation as a result of malabsorption. Parenteral nutrition should be considered and initiated accordingly.
- Clinical status, as well as BCAA levels, should be closely monitored in MSUD patients.
